# Vascular risk and cognitive trajectories in cognitively normal older adults: A Bayesian approach

**DOI:** 10.1002/alz70860_104040

**Published:** 2025-12-23

**Authors:** Meghan H Lewis, Christopher A. Gravel, Walter Swardfager, Peter P. Liu, Abhinav Sharma, Jodi D. Edwards

**Affiliations:** ^1^ University of Ottawa Heart Institute, Ottawa, ON, Canada; ^2^ University of Ottawa, Ottawa, ON, Canada; ^3^ McGill University, Montreal, QC, Canada; ^4^ Hurvitz Brain Sciences Program, Sunnybrook Research Institute, Toronto, ON, Canada; ^5^ Sunnybrook Research Institute, Toronto, ON, Canada; ^6^ Department of Pharmacology and Toxicology, University of Toronto, Toronto, ON, Canada; ^7^ ICES, Ottawa, ON, Canada

## Abstract

**Background:**

Cognitive decline poses a significant challenge to aging populations, with vascular risk factors—such as hypertension, diabetes, smoking, and hypercholesterolemia—playing a key role in its onset and progression. While prior studies using traditional statistical approaches, like linear mixed models, have demonstrated associations between vascular risk and cognitive decline, they often fail to fully account for uncertainty and variability in individual trajectories. This study aimed to estimate the influence of vascular risk factors on rates of cognitive decline in cognitively normal adults using a Bayesian statistical approach that incorporates prior knowledge and probabilistic reasoning.

**Method:**

We analyzed a cohort of cognitively normal adults aged 60 or older (*N* = 4,528) from the National Alzheimer's Coordinating Center (NACC) Uniform Data Set (UDS v3), followed for a median of 3.8 years. We constructed a measure of vascular risk based on individuals’ Framingham Risk Score (FRS), which incorporates factors such as age, systolic blood pressure, diabetes, smoking, and hypercholesterolemia. Cognitive decline was assessed as the change in Montreal Cognitive Assessment (MoCA) scores over time. Bayesian hierarchical models with random slopes and intercepts were fitted to estimate the relationship between vascular risk (high vs. low) and rates of cognitive decline, accounting for repeated measures within individuals.

**Result:**

Preliminary unadjusted findings indicate that individuals with high vascular risk (FRS ≥ 15 for males, ≥ 18 for females) had lower baseline MoCA scores (β = ‐1.12, 95% credible interval [‐1.33, ‐0.91]) compared to those with low risk (FRS ≤ 12 for males, ≤ 10 for females). High‐risk individuals also experienced a decline in MoCA scores over 6‐month intervals (β = ‐0.03, 95% credible interval [‐0.06, ‐0.00]), while the low‐risk group exhibited stable cognitive trajectories. Most variability in cognitive trajectories was explained by differences in baseline MoCA scores, with random slopes capturing modest variability in rates of change across individuals.

**Conclusion:**

These findings highlight the probabilistic relationship between vascular risk and cognitive decline, underscoring variability in individual trajectories. The Bayesian approach provides a robust framework for incorporating uncertainty and may help identify early risk phenotypes to better target interventions.

